# Cerebral blood flow variability in fibromyalgia syndrome: Relationships with emotional, clinical and functional variables

**DOI:** 10.1371/journal.pone.0204267

**Published:** 2018-09-20

**Authors:** Casandra I. Montoro, Stefan Duschek, Daniel Schuepbach, Miguel Gandarillas, Gustavo A. Reyes del Paso

**Affiliations:** 1 University of Jaén, Department of Psychology, Jaén, Spain; 2 UMIT—University for Health Sciences Medical Informatics and Technology, Hall in Tirol, Austria; 3 Klinikum am Weissenhof, Zentrum für Psychiatrie Weinsberg, Weinsberg, Germany; 4 University of Heidelberg, Department of General Psychiatry, Center of Psychosocial Medicine, Heidelberg, Germany; 5 Autonomous University of Madrid, Madrid, Spain; University of Würzburg, GERMANY

## Abstract

**Objective:**

This study analyzed variability in cerebral blood flow velocity (CBFV) and its association with emotional, clinical and functional variables and medication use in fibromyalgia syndrome (FMS).

**Methods:**

Using transcranial Doppler sonography, CBFV were bilaterally recorded in the anterior (ACA) and middle (MCA) cerebral arteries of 44 FMS patients and 31 healthy individuals during a 5-min resting period. Participants also completed questionnaires assessing pain, fatigue, insomnia, anxiety, depression and health-related quality of life (HRQoL).

**Results:**

Fast Fourier transformation revealed a spectral profile with four components: (1) a first very low frequency (VLF) component with the highest amplitude at 0.0024 Hz; (2) a second VLF component around 0.01-to-0.025 Hz; (3) a low frequency (LF) component from 0.075-to-0.11 Hz; and (4) a high frequency (HF) component with the lowest amplitude from 0.25-to-0.35 Hz. Compared to controls, FMS patients exhibited lower LF and HF CBFV variability in the MCAs (p < .005) and right ACA (p = .03), but higher variability at the first right MCA (p = .04) and left ACA (p = .005) VLF components. Emotional, clinical and functional variables were inversely related to LF and HF CBFV variability (r≥-.24, p≤.05). However, associations for the first VLF component were positive (r≥.28, p≤.05). While patients´ medication use was associated with lower CBFV variability, comorbid depression and anxiety disorders were unrelated to variability.

**Conclusions:**

Lower CBFV variability in the LF and HF ranges were observed in FMS, suggesting impaired coordination of cerebral regulatory systems. CBFV variability was differentially associated with clinical variables as a function of time-scale, with short-term variability being related to better clinical outcomes. CBFV variability analysis may be a promising tool to characterize FMS pathology and it impact on facets of HRQoL.

## Introduction

Fibromyalgia syndrome (FMS) is a chronic condition of widespread musculoskeletal pain accompanied by fatigue, sleep disturbance, depression, anxiety, cognitive deficits, etc. Though several factors can modulate the impact of the disease, FMS involves a severe reduction of psychosocial functioning and quality of life. In western countries, the prevalence of FMS is estimated at 2–4% in the general population, with women being predominantly affected [1] The etiology of FMS is largely unknown; however, it has been proposed that central nervous sensitization and deficient pain-inhibiting mechanisms play a key role in its pathophysiology [2, 3].

Based on this hypothesis, fMRI studies have analyzed cerebral blood flow (CBF) responses during painful stimulation in FMS. Their findings point towards increased activity in the neuromatrix of pain [4]. The investigation of CBF dynamics using transcranial Doppler sonography (TCD) has proven to be a useful complement to fMRI for analyses of pain-related CBF responses in FMS [5, 6]. This method provides continuous non-invasive measurement of CBF velocities (CBFV) in the basal cerebral arteries with high time resolution [7, 8]. The arteries most frequently investigated by TCD are the anterior cerebral artery (ACA), which supplies medial-anterior cerebral regions, including structures such as the ventromedial and orbital prefrontal cortex and superior parietal lobe; and the middle cerebral artery (MCA), which supplies lateral brain areas including the inferior parietal cortex and lateral sections of the frontal lobe [9]. The application of functional TCD during painful stimulation revealed a complex pattern of CBF modulations, where differences between patients and healthy individuals reflect augmented central nervous pain processing in FMS [5, 6, 10].

CBF shows spontaneous oscillations, which may be quantified through TCD analysis of beat-to-beat CBFV variability [11, 12]. Assessment of variability in hemodynamic variables like heart rate, blood pressure and CBF according to different time scales may provide insight into pathological mechanisms [13, 14]. Frequency domain analysis of resting CBFV variability in healthy individuals has revealed oscillations within specific frequency ranges, where variability in the very low frequency (VLF, < 0.04 Hz) and low frequency (LF, 0.04 to 0.15 Hz) ranges [11, 15, 16] showed higher spectral power than that observed in the high frequency (HF, 0.15 to 0.4 Hz) range [11, 13, 17–19].

Cerebral autoregulation plays an important role in the control of cerebral perfusion and CBF variability [15, 20]. Through adjustment of cerebral vascular resistance, cerebral autoregulation ensures relatively constant and adequate brain perfusion despite systemic blood pressure fluctuations [15, 20]. Precise control of CBF and cerebral perfusion is pivotal for the maintenance of normal brain function. It is therefore assumed that increased variability in hemodynamic variables, including CBF, is detrimental to brain function [13, 21], where autoregulatory mechanisms are sufficient to maintain constant CBF across a wide range of cerebral perfusion pressures [13, 21].

Although CBF variability may provide useful information regarding cardiovascular and cerebral health, the potential impact of increased variability in blood pressure and CBFV on clinical outcomes is not entirely clear. While some studies suggest a detrimental effect, others found a protective action. After analyzing this conflicting evidence, Rickards and Tzeng [13] concluded that the prognostic value of CBF variability depends on the specific time-scale considered. Long-term variations in blood pressure and CBF are linked with primary and secondary end-organ dysfunction, particularly in the context of secondary brain injury. By contrast, short-term blood pressure and CBF variability may fulfill a protective role, promoting optimal cerebral perfusion and oxygenation (even at low perfusion pressures), and preventing negative consequences of challenges like acute blood pressure changes, hypovolemia or cardiac arrest, and increasing tolerance to manipulations such as head-up tilt or body negative pressure [13]. Furthermore, short-term CBF variability has been associated with both baroreflex [22] and endothelial nitric oxide-mediated vasodilatation, and thus improved cerebral perfusion [23]. CBF variability, when maintained within physiological limits, may therefore be cerebro-protective and a marker of positive health status, reflecting the coordinated interplay of regulatory physiological systems [11, 13]. This is congruent with the well-known positive prognostic value of heart rate variability (HRV), and the inverse association of this parameter with various physical and psychological risk factors [14].

A number of previous studies demonstrated reduced HRV in FMS [24–27]. However, to the best of our knowledge, only one study has evaluated CBFV variability in FMS [28]. Rodríguez et al. [28] analyzed frequency, time-frequency and information theory features of the TCD raw signal, in addition to the so-called envelope curve of the TCD spectrum. While the TCD raw signal contains information on all of the blood cells moving at different velocities, the envelope curve is an already processed signal derived from the velocity of the fastest blood cells generating the highest Doppler shift [28, 29]. Power spectral analyses by Rodríguez et al. [28] showed a lower LF/HF ratio in the left ACA and both MCAs, and reduced spectral power at LF in the left MCA in FMS patients compared to controls. Lower values of the LF/HF ratio were associated with higher depression, trait-anxiety and pain intensity in the total sample. Moreover, the LF spectral power of the left MCA was inversely associated with pain severity. However, this study exclusively focused on the raw Doppler signal and envelope curve, and did not analyze variability of the mean (averaged velocity during the cardiac cycle) beat-to-beat CBFV index. The mean velocity measure shows the highest correlation with the absolute blood volume traveling through the artery [8].

The current study aimed to characterize mean beat-to-beat CBFV variability recorded via TCD during a resting state in FMS patients and healthy controls. Taking into account the research delineated above, we assumed that short-term CBFV variability would be positively linked to health status, and then our main hypotheses were as follows: (1) an overall reduction in CBFV spectral power variability in FMS patients than healthy individuals, especially in the LF and HF ranges; and (2) negative correlations between CBFV variability, especially in the LF and HF, and emotional (depression and anxiety) and clinical (pain severity, fatigue, insomnia) factors, as well as with health-related quality of life (HRQoL). Additionally, we explored possible differences in CBFV variability between FMS patients taking and not taking antidepressants, anxiolytics, and/or analgesic medication and between patients suffering and not suffering from comorbid depression and anxiety disorders.

## Methods

### Participants

Forty-four female FMS patients, aged between 20 and 63 years and recruited via the Fibromyalgia Association of Jaén, participated in the study. They were all examined by a rheumatologist and met the American College of Rheumatology criteria for FMS, which were used as the study inclusion criteria [1]. Exclusionary criteria comprised cardiovascular disease, metabolic abnormalities, neurological disorders, and severe somatic (e.g., cancer) or psychiatric (e.g., psychotic) diseases. The healthy control group included 31 women recruited from women's associations. They were comparable to the patients in terms of age and body mass index, as well as number of years of education (see Table 1) to control for their effects on HRQoL [30]. In addition to any kind of pain disorder and the lack of relatives suffering from FMS, the healthy group was subject to the same exclusionary criteria as the patients. Due to the higher prevalence of FMS in females than in males, and to avoid possible gender-related confounding factors, only females were included in the study.

**Table 1:** Means (±SD) of demographic, emotional and clinical variables in FMS patients and the control group. For medication use and comorbid depression and anxiety disorders the corresponding numbers of participants (percentage in brackets) are displayed.

	FMS patients	Control group	F or ӽ^2^	*p*
Age (years)	49.55 ± 8.42	47.03 ± 9.41	1.47	.229
Body mass index (kg/m^2^)	26.52 ± 3.54	25.38 ± 4.51	1.50	.224
Duration of education (years)	12.11 ± 3.20	12.90 ± 3.55	1.01	.318
Antidepressants (%)	21 (47.7%)	1 (3.2%)	18.53	< .0001
Anxiolytics (%)*	24 (54.5%)	10 (32.3%)	4.44	.035
Analgesics (%)	34 (77.3%)	5 (16.1%)	30.12	< .0001
Opioids (%)	16 (36.4%)	0 (0%)	15.12	< .0001
Depression (SCID) (%)	20 (45.5%)	4 (12.9%)	9.74	.002
Anxiety (SCID) (%)**	21 (47.7%)	4 (12.9%)	10.90	.001
Depression (BDI)	20.64±12.00	7.37±7.75	29.22	< .0001
Trait-anxiety (STAI-T)	35.36±8.88	19.38±9.91	53.50	< .0001
State-anxiety (STAI-S)	32.02±9.98	21.04±9.25	23.37	< .0001
Hypersomnia (OQSQ)	8.21±3.68	4.70±1.92	23.64	< .0001
Insomnia (OQSQ)	30.62±6.56	18.37±7.63	55.37	< .0001
Fatigue (FSS)	49.69±11.91	20.92±8.52	132.80	< .0001
Pain intensity (VAS)	3.60±.78	1.13±1.31	103.78	< .0001
Affective pain (MPQ)	5.98±4.82	1.27±1.63	27.34	< .0001
Total pain (MPQ)	53.76±32.29	18.23±11.90	30.81	< .0001
Physical HRQoL (SF-36)	38.44±9.04	65.79±4.33	243.77	< .0001
Mental HRQoL (SF-36)	33.79±9.18	54.41±8.92	93.93	< .0001

*Note*. Results of group comparisons are reported (univariate ANOVAs or ӽ^2^ tests). SCID = Structured Clinical Interview for Axis I Disorders of the Diagnostic and Statistical Manual for Mental Disorders; STAI-T = State-Trait Anxiety Inventory Trait Scale; STAI-S = State-Trait Anxiety Inventory State Scale; BDI = Beck Depression Inventory; FSS = Fatigue Severity Scale; OQSQ = Oviedo Quality of Sleep Questionnaire; VAS = Visual Analog Scale; MPQ = McGill Pain Questionnaire; HRQoL = Health-Related Quality of Life; SF-36 = Short-Form Health Survey

* The use of anxiolytics in healthy controls was sporadic and mainly related to sleep problems.

**Anxiety disorders comprised generalized anxiety disorder, panic disorder, phobias, and adjustment disorders.

### Recording and analysis of cerebral blood flow

Blood flow velocities were assessed by TCD via a digital Multi-Dop L2 (DWL (Elektronische Systeme GmbH, Sipplingen, Germany). Assessments were conducted bilaterally in the MCA and ACA. The recordings were obtained through the temporal bone windows using 2-MHz transducer probes. Following vessel identification, the probes were fixed to the head via a head harness. The MCA were insonated at a depth of 48–55 mm and the ACA at a depth of 60–70 mm. The spectral envelope curves of the Doppler signal were recorded at a rate of 100 samples per second.

**Fig 1:**
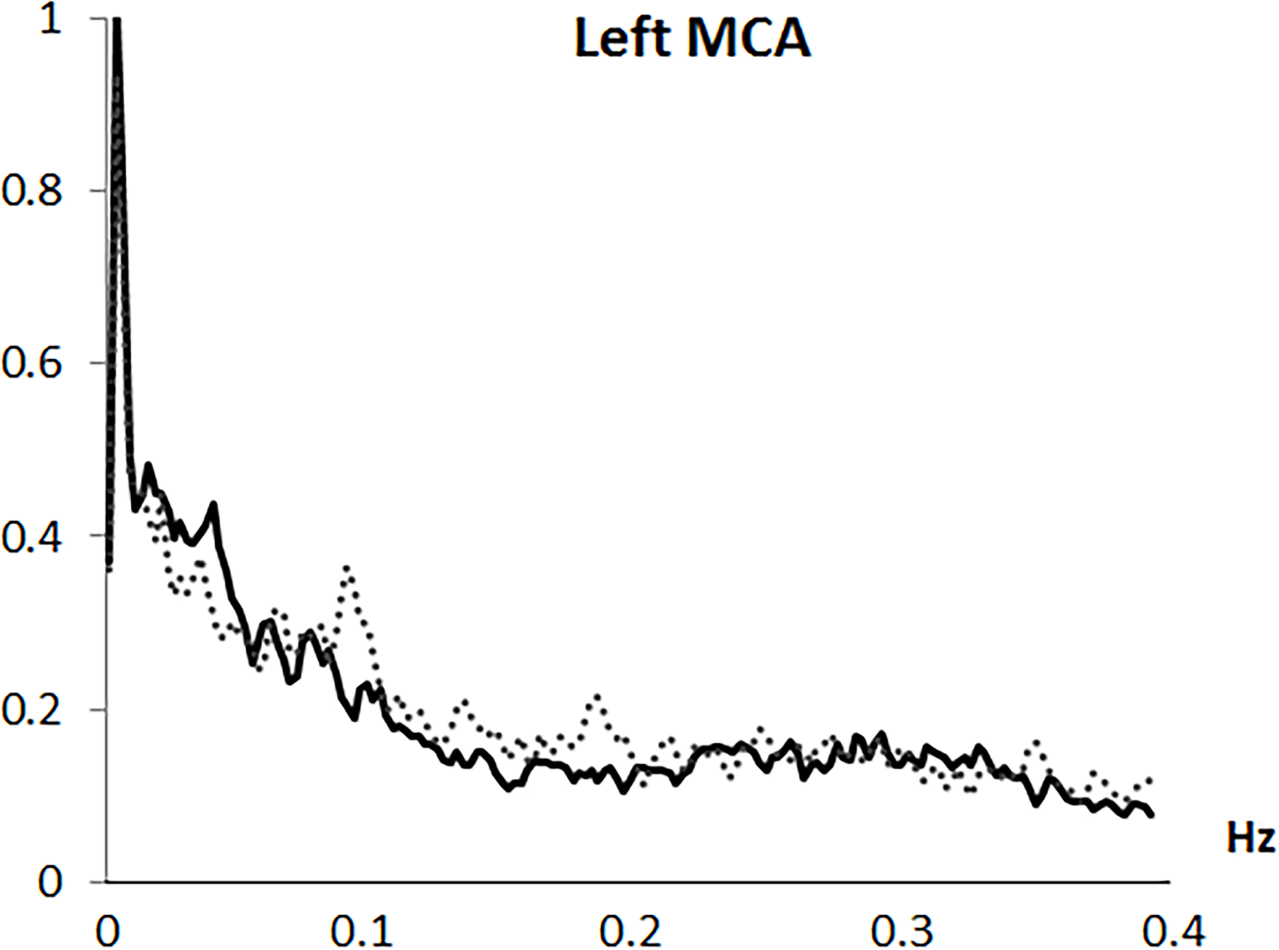
Frequency spectrum of cerebral blood flow velocity variability in the left middle artery (left MCA). Solid line represents fibromyalgia patients and dotted line represents control group.

**Fig 2:**
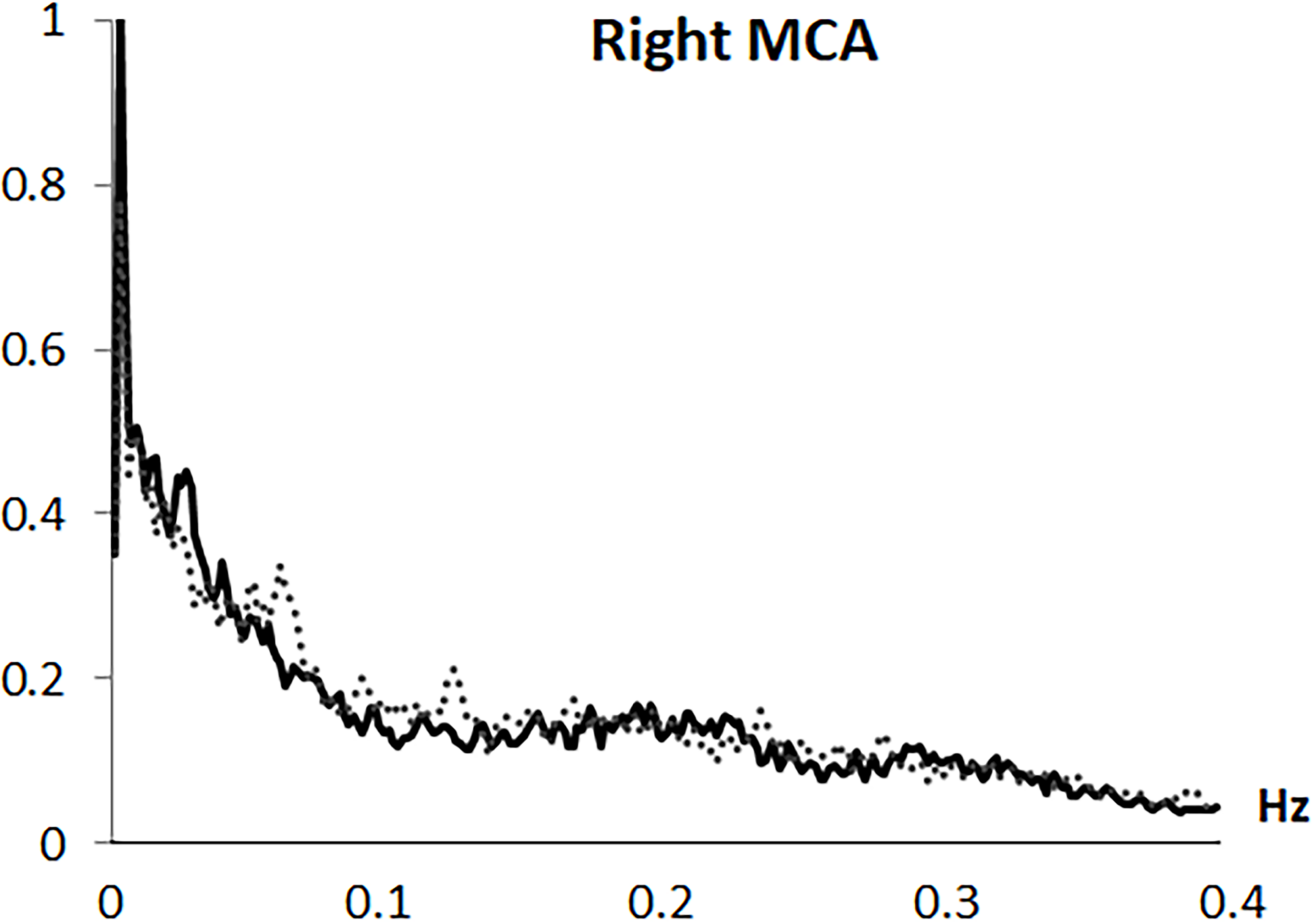
Frequency spectrum of cerebral blood flow velocity variability in the right middle artery (right MCA). Solid line represents fibromyalgia patients and dotted line represents control group.

**Fig 3:**
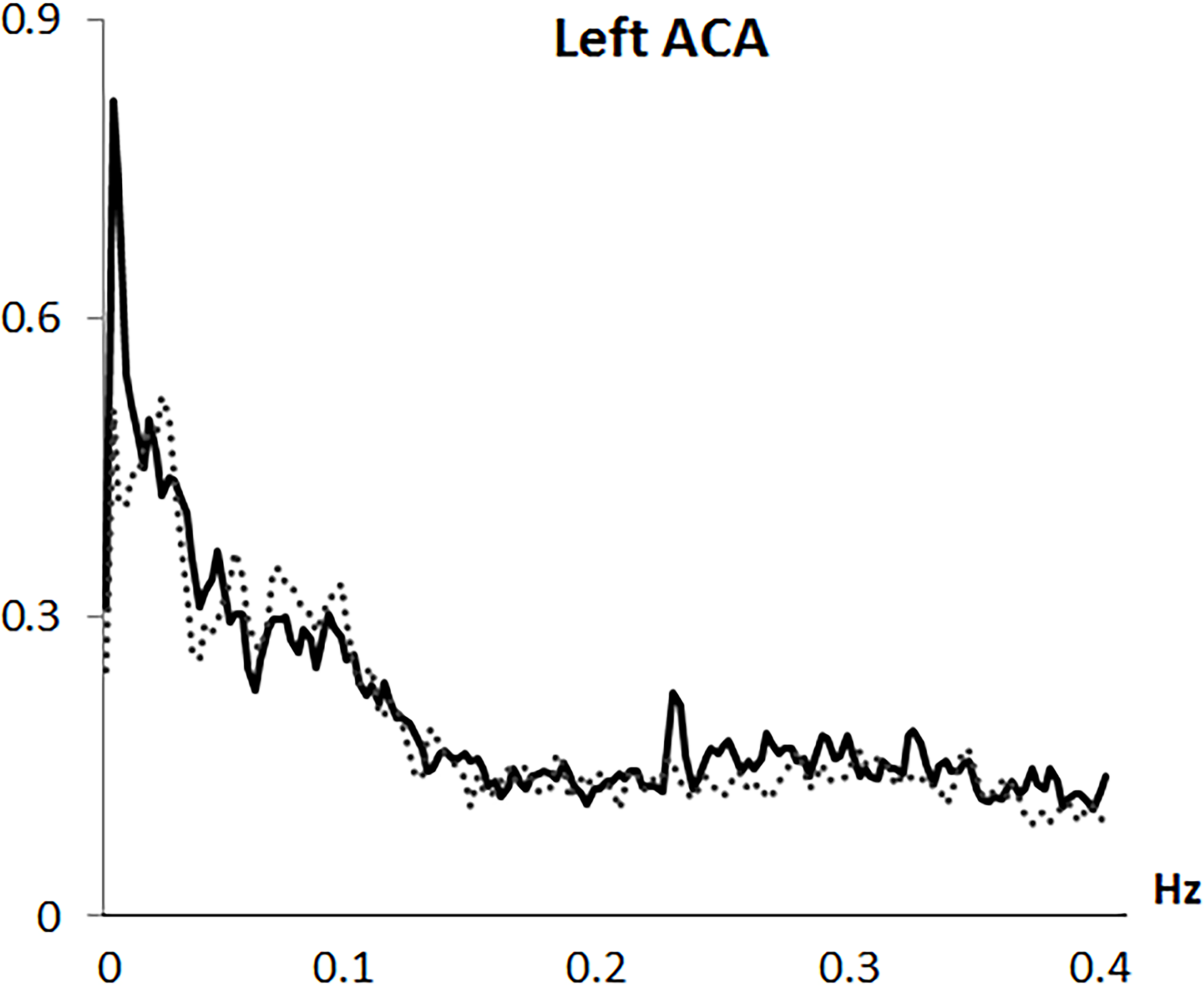
Frequency spectrum of cerebral blood flow velocity variability in the left anterior artery (left ACA). Solid line represents fibromyalgia patients and dotted line represents control group.

**Fig 4:**
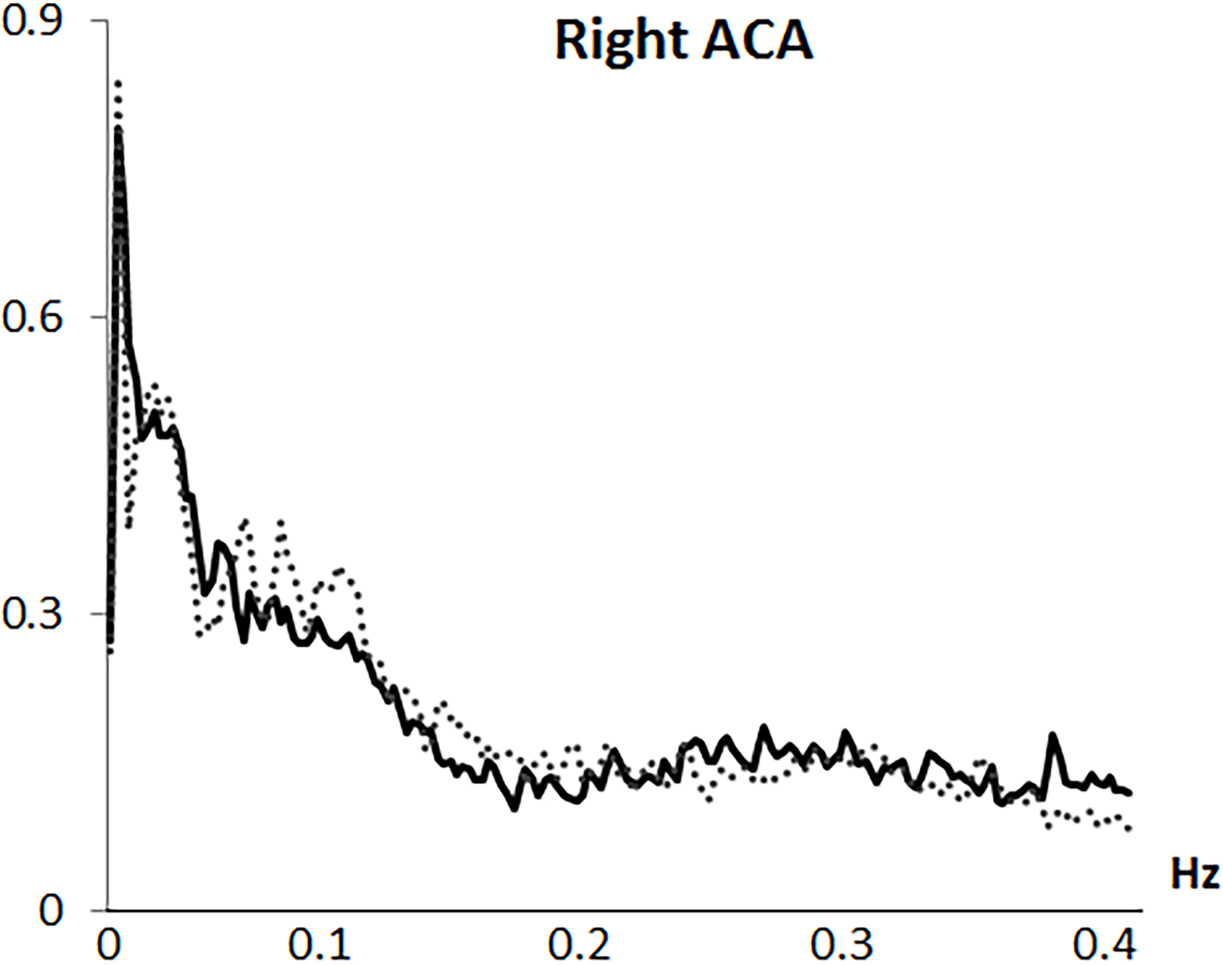
Frequency spectrum of cerebral blood flow velocity variability in the right anterior artery (right ACA). Solid line represents fibromyalgia patients and dotted line represents control group.

Analysis was based on the mean flow velocity index, as the averaged velocity within each cardiac cycle. Variability in mean CBFV was analyzed in the frequency domain by Fast Fourier transformation (FFT) based on a resampling at 4 Hz of the original 100 Hz recording, using AcqKnowledge 3.9.0 software (Biopac Systems Inc., Goleta, CA, USA). Through the algorithm described by Cooley & Tukey (1965) [31], the CBFV signal was divided into its frequency components (single sinusoidal oscillations). Before FFT computation, and to minimize spectral leakage, mean and slow trends in the data were removed. A shape for an anti-leakage termed “Hamming window function” was applied (see Claassen et al., 2016 [20]) and spectral power variability was computed in the frequency range between 0.0024 and 0.40 Hz, with a 0.0024 Hz resolution. A spectral profile with four main variability components was observed in all arteries and both study groups: (1) a first VLF component with a highest amplitude around 0.0024 Hz; (2) a second VLF component around 0.01 to 0.025 Hz; (3) a LF component with a frequency extension from 0.075 to 0.11 Hz; and (4) a HF component with the overall lowest amplitude and a frequency extension from 0.25 to 0.35 Hz (see Figs 1–4). Maximum peak variability values in each of these components, expressed in absolute units (cm/s^2^), were obtained.

### Procedure

The study was performed across two separate sessions that took place on different days. During the first session, a clinical psychologist recorded the patients’ clinical histories, medication use, and sociodemographic data via a semi-structured interview and confirmed that there were no violations of the exclusionary criteria. To evaluate possible mental disorders, the Structured Clinical Interview for Axis I Disorders of the Diagnostic and Statistical Manual for Mental Disorders [32] was employed. Symptoms of depression were assessed via the sum score of the Beck Depression Inventory (BDI) [33], and anxiety levels were quantified using the two scales of the Spanish version of the State-Trait Anxiety Inventory (STAI) [34]. Fatigue was assessed by a Spanish adaptation of the Fatigue Severity Scale (FFSS) [35]. Sleep quality was measured using the insomnia and hypersomnia indexes of the Oviedo Quality of Sleep Questionnaire (OQSQ) [36]. The McGill Pain Questionnaire (MPQ) [37] was applied for evaluation of clinical pain. The pain intensity index (visual analog scale, VAS), affective pain index and total pain index (given by the sum of sensory, affective and evaluative pain descriptors of the MPQ) were applied from this instrument. The Spanish adaptation of the Short-Form Health Survey (SF-36) [38] was applied for the assessment of HRQoL. The values of the eight subscales (i.e., functioning domains) were aggregated into the two general physical and mental SF-36 components using equations with the established weights for the Spanish population [39].

In the second session, after a 10-min period for adaptation to the laboratory, CBFV was recorded during a 5-min resting period. Recordings were performed in a seated position, where participants received instructions to sit still, not speak and relax, with their eyes open. Because simultaneous blood flow assessment in the MCA and ACA cannot be achieved with sufficient precision, this procedure was conducted twice, once for each pair of arteries. The artery assessment order (MCA or ACA first) was counterbalanced across participants. Inter-individual anatomical differences affect the possibility of successfully conducting TCD recordings [8]. Therefore, the number of participants with available data was different for each artery. The sample sizes were as follows: left MCA, 39 patients, 29 controls; right MCA, 39 patients, 29 controls; left ACA, 30 patients, 22 controls; and right ACA, 32 patients, 24 controls. Testing sessions were conducted starting at 10.30 a.m. and 5 p.m; half of the participants from the FMS and control groups were tested in the morning and the other half in the afternoon. This procedure aimed to control for circadian effects on CBFV variability. Participants were instructed to refrain from caffeine, alcohol, nicotine, any analgesic substance, and performance of vigorous exercise for 2 hours before the testing session. These factors were controlled since are known to affect central nervous activity and therefore CBFV [40–44]. Each participant gave written informed consent. The study protocol was approved by the Bioethics Committee of the University of Jaén.

### Statistical analysis

Group differences in CBFV variability in the four variability components were analyzed by univariate ANOVAs. Comparisons of CBFV variability between hemispheres were conducted using t-tests for related samples. Analysis of potential effects due to medication use and comorbid psychiatric disorders were analyzed by univariate ANOVAs comparing CBFV variability between FMS patients using and not using antidepressants, anxiolytics, analgesics, and opiates and FMS patients suffering and not suffering from depression and anxiety disorders. Effect sizes are indicated by adjusted eta squared ($\eta_{p}^{2}$). Associations between CBFV variability and questionnaire scale scores were quantified in two steps in the total sample: firstly, by Pearson correlations (two-tailed) and secondly by multiple stepwise regression analysis. CBFV variability in each of the components and arteries was taken as dependent variable (in separate analyses); questionnaires variables that showed significant associations with CBFV variability in the previous correlation analysis were used as predictors. These regression analyses provided an adjusted (by degrees of freedom) R^2^, used to index the predictive capacity of the model, and standardized β coefficients, representing the slope of the regression line. Alpha level was set at 5% in all analyses.

## Results

### Group differences in emotional, clinical and functional variables

Emotional, clinical and functional data in the two groups are summarized in Table 1. The FMS group showed higher levels of depression, anxiety, hypersomnia, insomnia, fatigue and pain severity than controls, but lower levels of physical and mental HRQoL. FMS patients also displayed higher prevalence of depression and anxiety disorders and more frequent use of the four medications considered.

### Group differences in mean CBFV variability

In both MCAs, lower LF and HF variability was found in FMS patients than healthy participants. However, variability in the first VLF component of the right MCA was greater in FMS patients. In the left ACA, greater variability in the first VLF component, but lower variability in the second VLF component, was seen in patients. Finally, for the right ACA, lower LF variability was observed in FMS patients than healthy participants (see Table 2). Regarding comparisons between arteries, no differences arose between left and right MCA or ACA, in either frequency component or group (all t ≤ 1.88, all p ≥ .070 for all analyses).

**Table 2:** Means (±SD) of variability in blood flow velocity (cm/s^2^) in the three frequency bands in FMS patients and controls. Results of the group comparison are also displayed.

Cerebral artery	Frequency Component	Mean±SD		*F*	*p*
		*FMS patients*	*Control group*		
Left MCA	VLF (1)	1.01±0.68	0.93±0.64	.25	.62
	VLF (2)	0.50±0.33	0.46±0.27	.23	.63
	LF	0.20±0.14	0.37±0.19	15.78	<.0001
	HF	0.12±0.09	0.21±0.15	11.27	.001
Right MCA	VLF (1)	1.06±0.65	0.78±0.34	4.38	.04
	VLF (2)	0.52±0.29	0.45±0.31	1.03	.32
	LF	0.21±0.15	0.34±0.19	8.72	.004
	HF	0.13±0.10	0.21±0.14	8.44	.005
Left ACA	VLF (1)	.83±0.36	0.52±0.40	8.44	.005
	VLF (2)	0.41±0.21	0.55±0.25	4.66	.03
	LF	0.28±.20	0.34±0.22	.80	.38
	HF	0.22±.27	0.15±0.10	1.22	.28
Right ACA	VLF (1)	0.79±0.35	0.87±0.74	.32	.58
	VLF (2)	0.48±0.30	0.55±0.33	.60	.44
	LF	0.29±0.16	0.40±0.22	5.08	.03
	HF	0.14±0.12	0.18±0.11	1.60	.21

*Note*: VLF = very low frequency; LF = low frequency; HF = high frequency; MCA = middle cerebral artery; ACA = anterior cerebral artery; (1) = first component; (2) = second component.

### Differences in mean CBFV variability as a function of medication use and psychiatric comorbidity in FMS patients

FMS patients taking antidepressants compared with those not taking antidepressants exhibited lower left LF MCA variability (0.16 ± .08 vs. 0.26 ± 0.18 cm/s^2^; F(1,37) = 5.47, p = .025, $\eta_{p}^{2}$ = .13). FMS patients using anxiolytics compared with those not using this medication showed lower LF left ACA variability (0.22 ± 0.14 vs. 0.41 ± 0.23 cm/s^2^; F(1,27) = 7.95, p = .009, $\eta_{p}^{2}$ = .23) and higher LF right ACA variability (0.33 ± 0.14 vs. 0.22 ± 0.12 cm/s^2^; F(1,29) = 5.86, p = .022, $\eta_{p}^{2}$ = .17). FMS patients taking analgesics compared with those not taking these drugs exhibited lower left ACA variability in the second VLF component (0.36 ± 0.14 vs. 0.52 ± 0.24 cm/s^2^; F(1,27) = 4.74, p = .039, $\eta_{p}^{2}$ = .15) and in the LF component (0.24 ± 0.16 vs. 0.52 ± 0.20 cm/s^2^; F(1,27) = 13.28, p = .001, $\eta_{p}^{2}$ = .34). Patients using opioids displayed lower LF right MCA variability than patients not using this medication (0.15 ± 0.10 vs. 0.26 ± 0.17 cm/s^2^; F (1,37) = 4.55, p = .040, $\eta_{p}^{2}$ = .11).

Comparisons of CBFV variability between patients suffering and not suffering from comorbid depression and anxiety disorders revealed no significant differences in any of the variability components (F ≤ 2.52, p ≥ 0.05, $\eta_{p}^{2}$ ≤ .07 in all analyses).

### Associations between mean CBFV variability and emotional, clinical and functional variables

Pearson correlations between CBFV variability and BDI and STAI scores in the total sample are displayed in Table 3. The BDI score correlated positively with variability in the first VLF component of the left ACA, but negatively with the second VLF and HF components of the right ACA. The STAI-S score correlated negatively with LF and HF MCA variability (both hemispheres), right LF and HF ACA variability, and left ACA second VLF component variability; however this score correlated positively with variability in the second VLF component of the right MCA. The STAI-T score correlated negatively with LF (left MCA and right ACA) and HF (both MCAs and right ACA) variability.

**Table 3:** Pearson correlations (coefficient r) between CBFV variability in the three frequency bands, and depression (BDI) and anxiety (STAI) in the total sample.

		*Depression* (BDI)	*State-anxiety* (STAI-S)	*Trait-anxiety* (STAI-T)
Left MCA	VLF (1)	-.08	.14	-.08
	VLF (2)	.02	.07	.05
	LF	-.20	-.38**	-.35**
	HF	-.23	-.31*	-.28*
Right MCA	VLF (1)	.06	.21	.03
	VLF (2)	.09	.28*	.21
	LF	-.06	-.37**	-.22
	HF	-.18	-.36**	-.26*
Left ACA	VLF (1)	.35**	.18	.27
	VLF (2)	-.16	-.33*	-.18
	LF	**-**.20	-.19	-.26
	HF	.08	.02	.04
Right ACA	VLF (1)	-.06	.11	-.10
	VLF (2)	-.32*	-.12	-.12
	LF	-.21	-.29*	-.37**
	HF	-.36**	-.41**	-.34*

*p < .05

**p < .01

*Note*: VLF = very low frequency; LF = low frequency; HF = high frequency; MCA = middle cerebral artery; ACA = anterior cerebral artery; (1) = first component, (2) = second component; BDI = Beck Depression Inventory; STAI-T = State-Trait Anxiety Inventory Trait Scale; STAI-S = State-Trait Anxiety Inventory State Scale

Table 4 presents the Pearson correlations between CBFV variability, OQSQ and FFSS scores in the total sample. The OQSQ Insomnia score correlated negatively with the second VLF left ACA variability component, and with LF and HF left MCA variability. The Hypersomnia score correlated negatively with the left LF and right HF MCA variability components, but positively with the second VLF MCA variability component (both hemispheres). Moreover, the FFSS score correlated negatively with variability in the second VLF left ACA component, LF and HF MCA (both hemispheres) and right LF ACA variability.

**Table 4:** Pearson correlations (coefficient r) between mean CBFV variability in the three frequency bands, sleep parameters (OQOS) and fatigue (FFSS) in the total sample.

		*Insomnia* (OQSQ)	*Hypersomnia* (OQSQ)	*Fatigue* (FFSS)
Left MCA	VLF (1)	.08	.15	.05
	VLF (2)	.11	.26*	.08
	LF	-.35**	-.34**	-.37**
	HF	-.26*	-.23	-.36**
Right MCA	VLF (1)	.09	.19	.18
	VLF (2)	.24	.27*	.17
	LF	-.21	-.20	-.24*
	HF	-.23	-.29*	-.33**
Left ACA	VLF (1)	.26	.18	.23
	VLF (2)	-.37**	-.22	-.27*
	LF	-.06	.01	-.03
	HF	.10	-.06	.09
Right ACA	VLF (1)	.02	.01	-.04
	VLF (2)	.09	.08	-.01
	LF	-.18	-.13	-.31*
	HF	-.03	.01	.22

*p < .05

**p < .01

*Note*: MCA = middle cerebral artery; ACA = anterior cerebral artery; VLF = very low frequency; LF = low frequency; HF = high frequency; (1) = frst component, (2) = second component; OQSQ = Oviedo Quality of Sleep Questionnaire; FSS = Fatigue Severity Scale

Table 5 shows the Pearson correlations between CBFV variability and MPQ scores in the total sample. The Pain Intensity score correlated negatively with LF and HF MCA (both hemispheres) and the second VLF left ACA component. The Total Pain score correlated negatively with LF left MCA, and the Affective Pain score correlated negatively with variability in LF left MCA variability but positively with variability in the first VLF right MCA and left ACA component.

**Table 5:** Person correlations (coefficient r) between mean CBFV variability in the three frequency bands and clinical pain (MPQ) in the total sample.

		Pain intensity (MPQ)	Affective pain (MPQ)	Total pain (MPQ)
Left MCA	VLF (1)	.04	.07	.03
	VLF (2)	.09	.15	.18
	LF	-.44**	-.29*	-.28*
	HF	-.36**	-.18	-.18
Right MCA	VLF (1)	.21	.31**	.21
	VLF (2)	.12	.20	.24
	LF	-.30*	-.14	-.14
	HF	-.32**	-.24	-.23
Left ACA	VLF (1)	.21	.28*	.27
	VLF (2)	-.40**	-.15	-.23
	LF	-.17	-.22	-.16
	HF	.12	.02	.03
Right ACA	VLF (1)	-.13	-.11	-.17
	VLF (2)	-.15	-.11	-.11
	LF	-.26	-.06	-.16
	HF	-.16	-.19	-.19

*p < .05

**p < .01

Note: MCA = middle cerebral artery; ACA = anterior cerebral artery; VLF = very low frequency; LF = low frequency; HF = high frequency; (1) = first component, (2) = second component; MPQ = McGill Pain Questionnaire.

Pearson correlations between CBFV variability and general SF-36 score in the total sample are displayed in Table 6. The physical HRQoL score correlated positively with LF and HF MCA (both hemispheres), HF right ACA, and the second VLF left ACA component, but negatively with variability in the first VLF left ACA component. The mental HRQoL score correlated positively with LF and HF MCA (both hemispheres), LF ACA (both hemispheres) and HF right ACA, but negatively with variability in the second VLF right MCA and first VLF ACA left components.

**Table 6:** Pearson correlations (coefficient r) between mean CBFV variability in the three frequency bands and health-related quality of live (SF-36) in the total sample.

		Physical HRQoL (SF-36)	Mental HRQoL (SF-36)
Left MCA	VLF (1)	-.01	.03
	VLF (2)	-.05	-.10
	LF	.44**	.40**
	HF	.39**	.41**
Right MCA	VLF (1)	-.12	-.12
	VLF (2)	-.14	-.28*
	LF	.33**	.28**
	HF	.35**	.32**
Left ACA	VLF (1)	-.35**	-.32*
	VLF (2)	.27*	.20
	LF	.15	.37**
	HF	-.11	-.10
Right ACA	VLF (1)	.02	.15
	VLF (2)	.14	.12
	LF	.24	.31*
	HF	.27*	.38**

*p < .05

**p < .01

Note: MCA = middle cerebral artery; ACA = anterior cerebral artery; VLF = very low frequency; LF = low frequency; HF = high frequency; (1) = first component, (2) = second component; HRQoL = Health-Related Quality of Life; SF-36 = Short-Form Health Survey.

Significant results of the multiple regression analyses for the prediction of CBFV variability by the questionnaire scores are summarized in Table 7. Concerning the left MCA, Hypersomnia (OQSQ) positively predicted the second VLF component and the Physical and Mental HRQoL scores (SF-36) positively predicted LF and HF variability. No significant predictors arose for the first VLF component. Regarding the right MCA, Affective Pain (MPQ) positively predicted the first VLF component, the Mental HRQoL score (SF-36) negatively predicted the second VLF component and State-Anxiety (STAI-S) negatively predicted the LF and HF components. Concerning the left ACA, Depression (BDI) positively predicted the first VLF component and Pain Intensity (MPQ) negatively predicted the second VLF component. Moreover, the Mental HRQoL score (SF-36) positively predicted LF variability. No significant predictors arose for HF variability. For the right ACA, Depression (BDI) negatively predicted the second VLF component and Trait-Anxiety (STAI-T) and State-Anxiety (STAI-S) negatively predicted LF and HF variability. No significant predictors were seen for the first VLF component.

**Table 7:** Results of multiple regression analysis for the prediction of CBFV variability in the left and right MCA and ACA.

Dependent variables		Predictors	stand. *β*	adj. *r*^*2*^	*t*	*P*
Left MCA	VLF (2)	Hypersomnia (OQSQ)	.26	.06	2.21	.031
	LF	Physical HRQoL (SF-36)	.44	.18	3.97	<.0001
	HF	Mental HRQoL (SF-36)	.41	.15	3.61	.001
Right MCA	VLF (1)	Affective Pain (MPQ)(MPQ)	.31	.08	2.66	.010
	VLF (2)	Mental HRQoL (SF-36)	-.28	.06	-2.35	.022
	LF	State-Anxiety (STAI-S)	-.37	.13	-3.25	.002
	HF	State-Anxiety (STAI-S)	-.36	.12	-3.17	.002
Left ACA	VLF (1)	Depression (BDI)	.35	.011	2.68	.010
	VLF (2)	Pain intensity (MPQ)	-.40	.14	-3.04	.004
	LF	Mental HRQoL (SF-36)	.37	.12	2.79	.007
Right ACA	VLF (2)	Depression (BDI)	-.32	.09	-2.48	.016
	LF	Trait-Anxiety (STAI-T)	-.37	.12	-2.90	.005
	HF	State-Anxiety (STAI-S)	-.41	.15	-3.31	.002

Note: MCA = middle cerebral artery; ACA = anterior cerebral artery; VLF = very low frequency; LF = low frequency; HF = high frequency; (1) = first component, (2) = second component; SF-36 = Short-Form Health Survey; HRQoL = Health-Related Quality of Life; MPQ = McGill Pain Questionnaire; OQSQ = Oviedo Quality of Sleep Questionnaire; STAI-S = State-Trait Anxiety Inventory State Scale; STAI-T = State-Trait Anxiety Inventory Trait Scale; BDI = Beck Depression Inventory.

## Discussion

This study, for the first time, characterized mean beat-to-beat CBFV variability in the MCA and ACA during resting state in FMS, and its linkage with emotional, clinical and functional factors. Frequency domain analysis revealed a spectral profile with four main variability components, similar to those previously observed in healthy individuals: a first VLF component with a highest amplitude at 0.0024 Hz, a second VLF component ranging between 0.01 and 0.025 Hz, a LF component between 0.075 and 0.11 Hz, and a HF component between 0.25 and 0.35 Hz (which had the lowest amplitude and greatest extension) [11, 17–19].

The analysis conducted in the entire frequency spectrum indicated substantial differences in CBFV variability between FMS patients and healthy individuals. In the first VLF component, these differences included higher CBFV variability in the right MCA and left ACA in patients than controls. However, lower CBFV variability in the second VLF component, LF and HF bands was seen in patients for the MCA and ACA of both hemispheres. More specifically, lower variability in the LF and HF bands for the MCA (both hemispheres), and in the second VLF component (left hemisphere) and LF (right hemisphere) bands for the ACA, arose in FMS. The lower LF MCA variability in FMS patients is congruent with the previous finding of Rodríguez et al. [28]. However, contrary to their study, the lower CBFV variability was not restricted to the LF left MCA, but also arose in other vessels and frequencies. Also, as stated above, higher CBFV variability in the first VLF component was observed in FMS patients. The discordance may be explained by differences between both studies regarding the processing of the TCD data, i.e. raw signal and envelope curve in Rodriguez et al. [28] versus mean beat-to-beat CBFV index in the present study; and the variability parameters obtained, i.e. the integral over specific bands (absolute power in the LF and HF ranges) and the ratio between LF to HF power in Rodriguez et al. [28] versus maximum peak value of each frequency band extracted by FFT in our study. It is evident that different CBFV variability parameters may reflect different underlying physiological mechanisms [45].

Resting variability in physiological systems has been considered as a marker of physical and mental health, being associated with favorable health status and positive prognosis [46]. Variability in non-linear systems reflects the ability to flexibility and rapidly cope with uncertain and changing environments [47]. Recent findings suggest that this also holds true for CBFV variability [13]. The reduced overall resting CBFV in FMS patients observed in our study is in accordance with this notion. In the cardiac domain, low HRV has been associated with risk factors such as stress, sedentary lifestyle, smoking, obesity, hypertension, hostility, depression and anxiety [14]. Consistently, overall HRV, as well as HRV within each of the three frequency bands, is lower in FMS patients than in healthy individuals [26, 27]. Overall decreased variability in cardiovascular signals in FMS may be interpreted as indicative of reduced autoregulatory capacity and deficient coordination among physiological control mechanisms [26, 27, 48]. Considering this, the lower CBFV variability seen in FMS might reflect reduced coordination and interplay among cerebral regulatory systems. Unlike the lower overall variability observed in FMS, specific variability in the first VLF component was greater in FMS patients than in healthy participants in the right MCA and left ACA. This VLF peak was observed at 0.0024 Hz, a frequency commonly referred to as ultra-low (see below).

Concerning the associations between CBFV variability and affective variables, questionnaire scores denoting aversive emotional states (depression and anxiety) were mainly inversely associated with CBFV variability. Both depression (BDI) and state and trait anxiety (STAI) correlated negatively with the second VLF, LF and HF variability components, especially in the right ACA. However, the BDI score was positively associated with variability in the first VLF component of the left ACA, and the STAI state score correlated positively with variability in the second VLF component of the right MCA.

The linkage of depression and anxiety levels with lower CBFV variability specifically in the right (and not left) ACA may be understood in the context of emotion-related brain asymmetry and the effect of mental load on CBFV variability. According to R.J. Davidson´s research on emotion-related brain asymmetry, frontal regions are differential involved in emotion processing [49]. Emotions that entail approach behavior (i.e., positive emotions) are accompanied by greater activation of left frontal regions, while emotions entailing withdrawal behavior (i.e., negative emotions) are associated with right hemispherical dominance [50, 51]. These right anterior areas are also associated with the emotional and cognitive processing of pain in FMS [9] and may be expected to be more active in individuals with high levels of negative affectivity [50, 51]. It has been shown that mental load increases CBF [11, 52–54] but reduces CBFV variability [11]. In a study of healthy individuals, the observed reduction in CBFV variability during arithmetic processing correlated positively with task performance, supporting its linkage with mental load [11] (i.e., more activity in the cerebral region irrigated by an artery is associated with a reduction in CBFV variability). Mental effort, emotional load and stress are associated with transient inhibition of autoregulatory control and homeostatic mechanisms, leading to decreased variability in physiological variables. These short-term responses support the organisms´ adjustment in order to successfully cope with situational challenges. This has been extensively analyzed in the field of heart rate variability [55, 56]. Within this framework, if greater activation of anterior right structures (related to negative affective states) in individuals with higher negative affect occurs, inverse associations between depression and anxiety scores and CBFV variability in the right ACA would be expected.

Regarding the connection between CBFV variability and clinical variables, insomnia hypersomnia and fatigue were overall inversely associated with variability in the second VLF, LF and HF components. However, hypersomnia was positively related to second component VLF MCA variability. Greater pain intensity (VAS), total and affective pain scores (MPQ) were associated with lower overall CBFV variability. In contrast, affective pain correlated positively with variability in the first VLF right MCA and left ACA component. Regarding the linkage between CBFV variability and functioning, SF-36 scores of physical and mental HRQoL were positively associated with second VLF, LF and HF variability components in both arteries and hemispheres. However, CBFV variability in the first VLF left ACA component was negatively associated with both SF-36 scores, and the mental score was also negatively associated with variability in the second VLF right MCA component.

The proposal of Rickards and Tzeng [13], of differentiating between detrimental *versus* protective roles of blood pressure and CBF variability according to timescale, may be instructive in interpreting the above patterns of associations. In addition to the enhanced first VLF component observed in patients´ right MCA and left ACA, the correlations with emotional, clinical and functional variables suggest that CBFV variability in the ultra-low frequency range (< .008 Hz) may be related to an unfavorable health state, while variability at higher frequencies was associated with positive health conditions. Additionally, our results suggest that variability in the second VLF component might be at the border of this differential outcome, displaying both negative and positive associations as a function of particular symptoms. In explaining the inverse associations of the first VLF component with health outcomes, it has been proposed that CBFV oscillations at lower frequency ranges are associated with myogenic activity and pathological changes in vascular properties. Specially, increased oscillations at lower frequencies in clinical conditions can be a marker of decreased arterial compliance, which may in turn result in a reduced capacity of the vasculature to buffer oscillatory changes in blood pressure [57, 58]. This interpretation accords with previous observations of differences in VLF CBF oscillations between healthy individuals and patients suffering from Alzheimer, migraine or carotid artery obstruction [17, 59, 60].

The patients´ use of antidepressant, anxiolytic, analgesic and opioid medications was associated with lower CBFV variability in the left LF MCA, left LF ACA, left second VLF ACA, and right LF MCA. There are two possible explanations for these differences. First, they may be due to a direct effect of drugs on CBFV variability, and second, they might reflect differences in the clinical state of patients. It may be that the most affected patients are those who take medications. The analysis performed regarding effects of psychiatric comorbidity did not reveal CBFV differences between FMS patients suffering and not suffering from depression or anxiety disorders. The finding that psychiatric comorbidity did not affect CBFV variability while the use of antidepressant and anxiolytic medication reduced it, suggests a direct effect of medication (at least concerning antidepressants and anxiolytics) on CBFV variability.

Some methodological limitations of the study have to be acknowledged. At first, the effect of medication on CBFV variability may have influenced the observed group differences. This problem is difficult to resolve, as most FMS patients take one or more kinds of drugs, making it extremely difficult to find patients free of medication. As the corresponding case numbers were too small for the comparison of subgroups of patients taking particular combinations of drugs, this approach did not seem accessible either. Additionally, a substantial part of our healthy participants were sporadically taking anxiolytics, which further complicates this issue. Another restriction pertains to sleep quality. As we did not evaluate the participants´ sleep quality during the night before the assessments, and given the observed negative associations between sleep problems and CBFV variability, effects of sleep disturbance on our observations cannot be ruled out.

## Conclusions

Our results showed overall lower VLF (second component, i.e., 0.01 to 0.025 Hz), LF and HF CBFV variability in the MCA and ACA of FMS patients than healthy individuals. However, the first VLF peak occurring at ultra-low frequencies (i.e., 0.0024 Hz) exhibited greater power in patients. The general inverse associations of emotional, clinical and functional impact variables with CBFV variability at LF and HF ranges support the notion that short-term CBFV variability at these frequencies might fulfill a protective role with respect to brain function and may be considered an index of positive health status. In contrast, CBFV variability at frequencies below 0.008 Hz appears to be associated with detrimental effects. Exploring CBFV variability in FMS may be a useful clinical tool to better define patients who are more severely affected by the disease and related negative affective states. Specifically, short-term beat-to-beat CBFV variability might indicate a positive prognosis in FMS.
