# Correction: Cerebral blood flow variability in fibromyalgia syndrome: Relationships with emotional, clinical and functional variables

**DOI:** 10.1371/journal.pone.0207004

**Published:** 2018-10-31

**Authors:** Casandra I. Montoro, Stefan Duschek, Daniel Schuepbach, Miguel A. Gandarillas, Gustavo A. Reyes del Paso

The fourth author’s name is missing the middle initial. The correct name is: Miguel A Gandarillas. The correct citation is: Montoro CI, Duschek S, Schuepbach D, Gandarillas MA, Reyes del Paso GA (2018) Cerebral blood flow variability in fibromyalgia syndrome: Relationships with emotional, clinical and functional variables. PLoS ONE 13(9): e0204267. https://doi.org/10.1371/journal.pone.0204267

The affiliation of the fourth author is listed incorrectly. The correct affiliation is 5: Autonomous University of Madrid, Department of Social Psychology and Methodology, Madrid, Spain.

In Table 1, “ӽ” is missing from the heading in the fourth column and the footnote. Please see the correct Table 1 here.

**Table 1:** Means (±SD) of demographic, emotional and clinical variables in FMS patients and the control group. For medication use and comorbid depression and anxiety disorders the corresponding numbers of participants (percentage in brackets) are displayed.

	FMS patients	Control group	F or ӽ^2^	*p*
Age (years)	49.55 ± 8.42	47.03 ± 9.41	1.47	.229
Body mass index (kg/m^2^)	26.52 ± 3.54	25.38 ± 4.51	1.50	.224
Duration of education (years)	12.11 ± 3.20	12.90 ± 3.55	1.01	.318
Antidepressants (%)	21 (47.7%)	1 (3.2%)	18.53	< .0001
Anxiolytics (%)*	24 (54.5%)	10 (32.3%)	4.44	.035
Analgesics (%)	34 (77.3%)	5 (16.1%)	30.12	< .0001
Opioids (%)	16 (36.4%)	0 (0%)	15.12	< .0001
Depression (SCID) (%)	20 (45.5%)	4 (12.9%)	9.74	.002
Anxiety (SCID) (%)**	21 (47.7%)	4 (12.9%)	10.90	.001
Depression (BDI)	20.64±12.00	7.37±7.75	29.22	< .0001
Trait-anxiety (STAI-T)	35.36±8.88	19.38±9.91	53.50	< .0001
State-anxiety (STAI-S)	32.02±9.98	21.04±9.25	23.37	< .0001
Hypersomnia (OQSQ)	8.21±3.68	4.70±1.92	23.64	< .0001
Insomnia (OQSQ)	30.62±6.56	18.37±7.63	55.37	< .0001
Fatigue (FSS)	49.69±11.91	20.92±8.52	132.80	< .0001
Pain intensity (VAS)	3.60±.78	1.13±1.31	103.78	< .0001
Affective pain (MPQ)	5.98±4.82	1.27±1.63	27.34	< .0001
Total pain (MPQ)	53.76±32.29	18.23±11.90	30.81	< .0001
Physical HRQoL (SF-36)	38.44±9.04	65.79±4.33	243.77	< .0001
Mental HRQoL (SF-36)	33.79±9.18	54.41±8.92	93.93	< .0001

*Note*. Results of group comparisons are reported (univariate ANOVAs or ӽ^2^ tests). SCID = Structured Clinical Interview for Axis I Disorders of the Diagnostic and Statistical Manual for Mental Disorders; STAI-T = State-Trait Anxiety Inventory Trait Scale; STAI-S = State-Trait Anxiety Inventory State Scale; BDI = Beck Depression Inventory; FSS = Fatigue Severity Scale; OQSQ = Oviedo Quality of Sleep Questionnaire; VAS = Visual Analog Scale; MPQ = McGill Pain Questionnaire; HRQoL = Health-Related Quality of Life; SF-36 = Short-Form Health Survey;

* The use of anxiolytics in healthy controls was sporadic and mainly related to sleep problems.

**Anxiety disorders comprised generalized anxiety disorder, panic disorder, phobias, and adjustment disorders.
